# Effects of mosquito resting site temperatures on the estimation of pathogen development rates in near-natural habitats in Germany

**DOI:** 10.1186/s13071-022-05505-2

**Published:** 2022-10-25

**Authors:** Felix Gregor Sauer, Ellen Kiel, Renke Lühken

**Affiliations:** 1 Arbovirus Ecology, Department of Arbovirology, Bernhard Nocht Institute for Tropical Medicine, Hamburg, Germany; 2 Aquatic Ecology and Nature Conservation, Carl Von Ossietzky University, Oldenburg, Germany

**Keywords:** Culicidae, Microclimate, Resting sites, Extrinsic incubation period

## Abstract

**Background:**

Environmental temperature is a key driver for the transmission risk of mosquito-borne pathogens. Epidemiological models usually relate to temperature data from standardized weather stations, but these data may not capture the relevant scale where mosquitoes experience environmental temperatures. As mosquitoes are assumed to spend most of their lifetime in resting sites, we analysed mosquito resting site patterns and the associated temperatures in dependence on the resting site type, resting site height and the surrounding land use.

**Methods:**

The study was conducted in 20 areas in near-natural habitats in Germany. Ten areas were studied in 2017, and another 10 in 2018. Each study area consisted of three sampling sites, where we collected mosquitoes and microclimatic data in artificial (= garden pop-up bags) and natural resting sites at three height levels between 0 and 6 m. Land use of the study sites was characterized as forest and meadows based on reclassified information of the CORINE (Coordination of Information on the Environment) Land Cover categories. The hourly resting site temperatures and the data from the nearest weather station of the German meteorological service were used to model the duration of the extrinsic incubation period (EIP) of mosquito-borne pathogens.

**Results:**

*Anopheles, Culex* and *Culiseta* preferred artificial resting sites, while *Aedes* were predominantly collect in natural resting sites. Around 90% of the mosquitoes were collected from resting sites below 2 m. The mosquito species composition did not differ significantly between forest and meadow sites. Mean resting site temperatures near the ground were approximately 0.8 °C lower than at a height of 4–6 m, which changed the predicted mean EIP up to 5 days at meadow and 2 days at forest sites. Compared with temperature data from standardized weather stations, the resting site temperatures near the ground would prolong the mean estimated EIP 4 days at forest sites and 2 days at meadow sites.

**Conclusions:**

The microclimate of mosquito resting sites differs from standardized meteorological data, which can influence the transmission of mosquito-borne pathogens. In a near-natural environment, colder temperatures at mosquitoes’ preferred resting sites near the ground would prolong the EIP of mosquito-borne pathogens relative to data from weather stations.

**Graphical Abstract:**

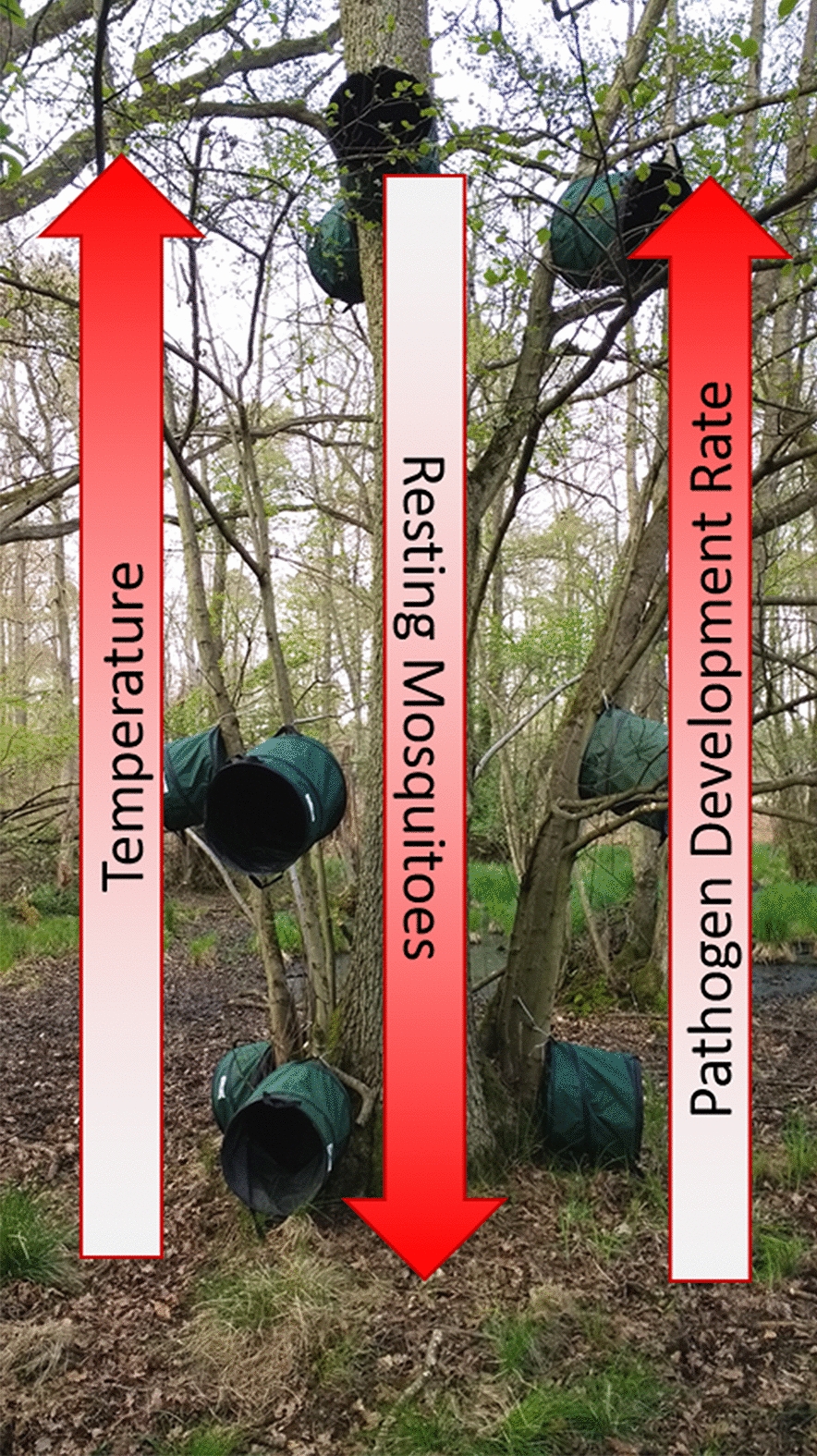

**Supplementary Information:**

The online version contains supplementary material available at 10.1186/s13071-022-05505-2.

## Background

Mosquitoes (Diptera: Culicidae) are the most important arthropod vector group. Transmission of mosquito-borne pathogens causes approximately 350 million infected humans and 500 thousand deaths per year [1]. Most cases are observed in tropical and subtropical regions. However, the global exchange of goods and passenger traffic in combination with a rapidly changing environment, e.g. caused by land use change and climate warming, promote the spread of invasive mosquito species and mosquito-borne pathogens [2, 3]. The emergence or re-emergence of several mosquito-borne pathogens in Europe has clearly documented the growing medical importance of vector-borne diseases. Regular outbreaks of West Nile virus (WNV) [4, 5] and chikungunya virus [6] have been recognized in Southern and South-East Europe. In Central Europe, ongoing Usutu virus circulation in birds [7–9] as well as human autochthonous cases of *Dirofilaria* [10] and WNV infections highlight the emerging risk also for Central Europe [11–13].

Temperature is among the most important drivers of the transmission risk of mosquito-borne pathogens. The body temperature of mosquitoes as ectothermic insects is equivalent to the ambient temperature [14]. Temperature strongly affects both the biological life history traits of mosquitoes (e.g. adult mortality and biting rates) and the pathogen development rate [15]. The inverse of the pathogen development rate is referred to as the extrinsic incubation period (EIP), i.e. the time it takes for a pathogen to develop or disseminate in a vector until it can be transmitted [16]. Each pathogen has a specific temperature-dependent development rate and temperature threshold preventing further development, e.g. 14–15 °C for WNV [17]. Accordingly, the integration of temperature in epidemiological models is crucial for assessing the spatial–temporal risk of mosquito-borne pathogen transmission. Temperature data for epidemiological models are usually obtained from weather stations [18] or remote sensing [19]. To provide unbiased meteorological measurements, the observational weather data are strictly standardized, e.g. weather stations shall be installed at freely exposed sites of at least 25 × 25 m without nearby buildings, trees or other structures [20]. The standardized nature of data collection from weather stations does allow for more direct comparison across geographic regions, but may not be sufficient for capturing the microclimate conditions important to mosquito and pathogen development [21].

With a high proportion of blood-engorged females in the resting site [22], resting site temperatures are expected to have a strong effect on the blood meal digestion periods and EIPs. The microclimatic temperatures in mosquito resting sites can be influenced by various small-scale (e.g. type of the microhabitat) and large-scale factors (e.g. surrounding land cover) [23, 24]. Furthermore, the active resting site selection is influenced by small-scale factors such as the type of microhabitat or the vertical height level [23, 25], and thus probably affect the temperature acting on the mosquitoes, which are considered to spend most of their life-time in resting sites [26]. Moreover, mosquito species composition is known to be affected by large-scale factors such as land cover [27]. Several studies have noted that the temperature in the potential habitats of mosquitoes can vary considerably from standardized meteorological data, resulting in large deviations in the predictions of the EIP [28–31]. However, these studies just measured temperature and humidity data in suspected resting sites of adult mosquitoes and did not include actual collections of mosquitoes. Thus, it remained unclear which particular microclimate is most relevant for mosquitoes. This knowledge gap can be closed by understanding mosquito resting site patterns.

In this study, we analysed the mosquito resting site patterns and the associated resting site temperatures with respect to their dependence on three different spatial factors: resting site type (artificial vs. natural), vertical height level between 0 and 6 m and land use category (forest vs. meadows). The study was conducted in different study areas in Germany and was focused on near-natural habitats. The aim was to perform an integrative analysis on the effects of the spatial factors on the microclimate of resting mosquito populations and to analyse the difference between resting site temperatures and standardized meteorological data on the calculation of the EIP of mosquito-borne pathogens to refine epidemiological models.

## Methods

### Study sites

**Fig. 1:**
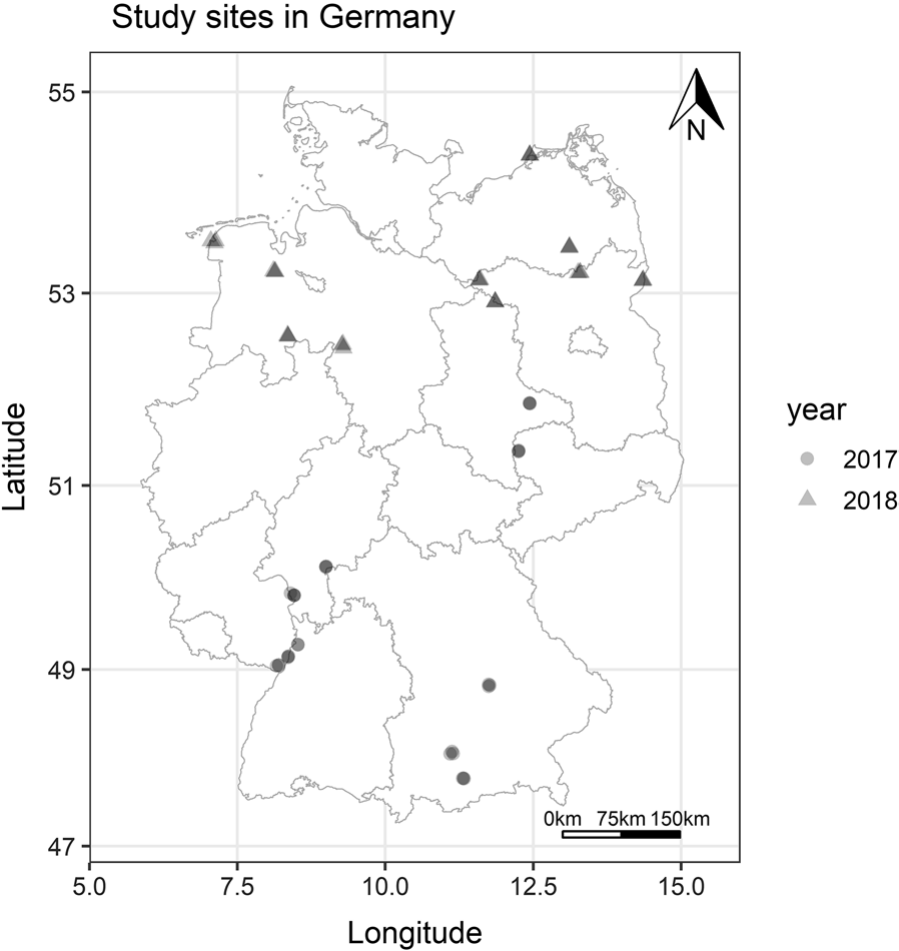
Study sites in Germany for both study years (circles: 2017, triangles: 2018)

The study was conducted at 20 study areas in Germany, including 10 studied in 2017 and another 10 in the subsequent year (Fig. 1). All study areas were selected based on their association with different types of near-natural wetlands, e.g. floodplains, swamp forest and reeds. Each study area consisted of three sampling sites, which were within 200 to 2000 m of each other. The three sampling sites in the near-natural study areas were placed in habitats with different vegetation, i.e. meadows vs. densely forested sampling sites, to cover a broad range of potential resting environments and microclimates.

### Mosquito collection

The sampling set-up was installed at the end of April in both years. In general, six potential natural resting habitats and nine artificial resting sites were sampled at each sampling site. Mosquitoes were collected from garden pop-up bags (Relaxdays GmbH, Halle, Germany; 76 l), which are known to be accepted as an artificial resting site by various mosquito species [25, 33]. Three garden pop-up bags were installed in different directions in one tree at three height levels (0 m, 2 m and 5 m). Five sampling sites could not be equipped with garden pop-up bags at heights greater than 2 m, because the trees on site were not high or robust enough to instal the garden pop-up bags at a height of 5 m. In addition, natural resting sites were sampled at two height levels (0–2 m and 2–6 m) parallel to the sampling the artificial resting sites. Mosquito sampling at three different heights did not seem appropriate for natural resting sites. Potential natural resting sites at an intermediate height level (1–2 m) were often not available due to season- and site-specific differences in the understory vegetation, in particular. In addition, resting mosquitoes can be disturbed and activated during sampling at natural resting sites, which would have hampered the sampling of natural resting sites close to each other, i.e. at 0–1 m and 1–2 m. Hence, we decided to differentiate between only two height levels in natural resting site habitats: 0–2 m (e.g. herb layer) and 2–6 m (e.g. tree branches). Each natural resting site habitat was sampled for 30 s and was located at a maximum distance of approximately 20 m from the tree equipped with the garden pop-up bags. We consider the maximum distance of 20 m between natural and artificial resting sites as easy to reach for a mosquito. Hence, the results obtained by natural and artificial resting site sampling can be directly compared, allowing conclusions about mosquito resting site preferences for the two different types of resting sites. In order to avoid any collectors’ bias, mosquito collection in resting habitats was exclusively conducted by one author (FGS). The collection was performed with hand-made aspirators similar to the model reported by Vazquez-Prokopec et al. [34], which is proven to perform equivalently to the Centers for Disease Control and Prevention Backpack Aspirator [35]. The hand-made aspirator could be equipped with a telescopic rod that can be extended up to 3.1 m. Resting mosquitoes were collected during three sampling periods. Each sampling period lasted 3 weeks, and sampling was conducted from mid-May to the beginning of June, mid -July to the beginning of August and in September. During the sampling periods, each study area was visited 2–3 times. The collected mosquitoes were transported in cooling boxes at − 18 °C until they were stored in a freezer (−20 °C) in the laboratory. Mosquitoes were identified by morphological characters to the lowest taxonomic level of certainty [36–38]. The feeding status of female mosquitoes was scored from one (unfed) to seven (eggs fully developed) following Detinova [39]. In the statistical analyses, the term “blood-fed” summarizes all females from freshly engorged to fully developed eggs.

### Microclimate data collection

At each study site, temperature loggers (HOBO Pendant logger UA-001–08, Onset Computer Corporation, USA) were installed in the vegetation at three height levels (0.3 m, 2 m and 5 m) in close proximity (< 10 m) to the tree equipped with garden pop-up bags. One additional logger recording temperature and relative humidity (Hobo Pro v2 U23, Onset Computer Corporation, USA) was placed in the south exposed garden pop-up bag at an intermediate height level (2 m) for each study site, to evaluate the temperature difference between artificial and natural resting sites. The microclimate data were logged hourly from May to the end of October in both study years. Thereby, a total of 120 loggers (90 temperature and 30 temperature and relative humidity loggers) were installed per study year.

### Analysis

The CORINE (Coordination of Information on the Environment) land cover (CLC) [40] of each sampling site was extracted to describe the surrounding land use in a 20 m buffer, i.e. the maximum distance between the mosquito collection sites and data loggers. CLC provides generalized classes of land use for Europe. Its nomenclature includes 44 classes with a minimum mapping unit of 25 hectares and a minimum width of 100 m [40]. Our 60 study sites included 12 different land use categories, which were reclassified into the two major habitats meadows and forests following the approach by Haider et al. [23]. The aggregated category “meadows” included complex cultivation patterns (CLC 242, *n* = 2), inland marshes (*n* = 1), land principally occupied by agriculture, with significant areas of natural vegetation (CLC 243, *n* = 7), natural grasslands (CLC 321, *n* = 3), non-irrigated arable land (CLC 211, *n* = 5), pastures (CLC 231, *n* = 12) and salt marshes (CLC 241, *n* = 1). The aggregated category “forest” included broad-leaved forest (CLC 311, *n* = 16), green urban area (CLC 141, *n* = 1), coniferous forest (CLC 312, *n* = 7), mixed forest (CLC 313, *n* = 5).

Hourly temperature, relative humidity, wind, solar radiation and precipitation were extracted from the nearest weather station of Germany’s national meteorological service (DWD, “Deutscher Wetterdienst”) for each study site. These standardized meteorological data were used to predict the microclimatic temperatures following the linear regression model by Haider et al. [28]:

$$\begin{aligned} temperature_{{micro}} = & temperature_{{DWD}} + temperature_{{DWD(t - 1)}} \\ & + solarradiation_{{DWD}} + windspeed_{{DWD}} \\ & + rel.humidity_{{DWD}} + monthweight\left( {May} \right) \\ & + monthweight\left( {June} \right) + monthweight\left( {July} \right) \\ & + monthweight\left( {August} \right) + monthweight\left( {September} \right) \\ & + monthweight\left( {October} \right) + timeofday\left( {hourly} \right) \\ & + \left( {solar*wind} \right) + \left( {precipation*humidity} \right) \\ & + \left( {wind*height} \right) + (solar*height) \\ \end{aligned}$$

Multiple linear regression was performed to predict the microclimate in the two habitats, forest and meadows, for both study years, respectively. The hourly temperature data for each logger and for the DWD weather stations were used to calculate the temperature-dependent EIP of three different mosquito-borne pathogens and the duration of the blood meal digestion period based on models retrieved from the literature (Table 1). In addition, the hourly difference between the EIP predictions based on the weather stations and based on the resting site temperatures were calculated and visualized. Therefore, due to the large data sets, we calculated the 99% confidence intervals, instead of the commonly used 95%, for visualization. Mosquito species composition for the different sites was analysed by means of a non-metric multidimensional scaling (NMDS). Subsequently, the effect of the land use categories forest and meadows on the species composition was analysed with a multivariate analysis of variance (MANOVA) using the *adonis* function in the R package “vegan” [41]. The extraction of land use information, the summary statistics of the microclimate and EIP data and the data visualization were conducted with the packages “raster”, “dplyr” and “ggplot2” in R [42–45].

**Table 1:** Temperature (T) dependent models to predict the extrinsic incubation period (EIP) of mosquito-borne pathogens and the blood meal digestion period (BMD)

Parameter	Equation	References
EIP *Dirofilaria immitis*	[(T-14)/130]/24	Ledesma and Harrington [46]
EIP *Plasmodium vivax*	[0.000126 × T × (T-14.244) × √(34.4-T)]/24	Cator et al. [29]
EIP West Nile virus	[−0.132 + 0.0092 × T)/24	Hartley et al. [17]
BMD	[(T-9.9)/36.5]/24	Lindsay et al. [32]

## Results

### Microclimate

The recorded temperatures in 2017 were lower than in 2018 (2017: mean = 16.02, 99% confidence interval ( ±) 0.02 °C, 2018: mean = 16.80 ± 0.02 °C). Accordingly, the relative humidity was higher in 2017 compared with 2018 (2017: mean = 83% ± 0.10%, 2018: mean = 77.97% ± 0.09%). In 2017, the maximum and minimum microclimatic temperature ranged from −5.13 °C to 49.22 °C and from −2.96 °C to 47.37 °C in 2018. Multiple linear regressions using the DWD data to predict the microclimatic temperatures explained 89% to 93% of the proportion of variance (R^2^). In both years, the model prediction accuracy was slightly higher for forest sites (in 2017: 93%, in 2018: 91%) compared with meadow sites (in 2017: 89%, in 2018: 89%) (see Additional file 1: Table S1 for all coefficients).

### Effects of the resting site type

Summarized for both study periods, the temperature in garden pop-up bags (mean = 16.37 °C ± 0.03 °C) was slightly lower than in the natural resting sites (mean = 16.47 °C ± 0.03 °C), both calculated based on data loggers installed at intermediate height level (1–2 m). The temperature difference between artificial and natural resting sites led to a mean difference in the calculated EIP of 0.64 days for WNV, 0.62 days for *Dirofilaria immitis,* 0.39 days for *Plasmodium vivax* and 0.08 days for the blood meal digestion period, indicating only a minor influence on epidemiological models, when measuring the temperature inside garden pop-up bags or directly in the natural resting sites.

The mosquito species composition in artificial and natural resting sites differed strongly. *Aedes* specimens dominated in natural resting sites (83%), but were less prominent in garden pop-up bags (8%). On the other hand, artificial resting sites were frequently used by *Culiseta* (68%) and *Anopheles* (8%). *Culiseta morsitans*/*fumipennis* was the dominant taxon in garden pop-up bags, accounting for almost 60% of the individuals, but comprised only 4% of the mosquitoes collected in natural resting sites. *Culex pipiens* s.l./*Cx. torrentium* was collected in similar numbers in garden pop-up bags and natural resting sites, while species from the subgenus *Neoculex*, namely, *Cx. territans* and *Cx. hortensis*, were collected in higher numbers by means of garden pop-up bags (see Table 2 for absolute number of collected specimens).

**Table 2:** Total number of collected mosquito taxa per sampling method. Taxa per genus are given in descending order based on the numbers of collected specimens

Genus	Mosquito taxon	Artificial resting sites			Natural resting sites		
		Female	Male	Sum	Female	Male	Sum
*Aedes*	*Ae. annulipes* group^a^	168	0	168	1246	28	1274
	*Ae. annulipes*^a^	0	11	11	0	558	558
	*Ae. cantans*^a^	0	6	6	0	230	230
	*Ae. vexans*	24	3	27	282	283	565
	*Ae. cinereus*	13	0	13	130	78	208
	*Ae. sticticus*	22	0	22	81	42	123
	*Ae. rusticus*	11	3	14	71	26	97
	*Ae. punctor*	9	2	11	42	45	87
	*Ae. flavescens*	0	0	0	13	38	51
	*Ae. communis*	6	3	9	17	13	30
	*Ae. rossicus*	1	0	1	5	1	6
	*Ae. caspius*	0	0	0	5	2	7
	*Ae. detritus*	1	1	2	0	3	3
	*Ae. cataphylla*	2	0	2	1	0	1
	*Ae. pullatus*	0	0	0	1	0	1
	*Ae. diantaeus*	0	0	0	1	0	1
*Anopheles*	*An. maculipennis* s.l.	106	129	235	2	3	5
	*An. claviger*	5	3	8	9	8	17
	*An. plumbeus*	16	13	29	2	0	2
*Coquillettidia*	*Cq. richiardii*	8	14	22	6	21	27
*Culiseta*	*Cs. morsitans*/*fumipennis*	1366	694	2060	98	55	153
	*Cs. annulata*/*subochrea*	139	128	267	12	29	41
	*Cs. alaskaensis*	0	0	0	0	1	1
	*Cs. glaphyroptera*	3	5	8	0	0	0
*Culex*	*Cx. pipiens* s.l./*Cx. torrentium*	179	132	311	81	129	210
	*Cx. territans*	102	40	142	17	19	36
	*Cx. hortensis*	7	4	11	0	0	0
	*Cx. modestus*	1	1	2	0	0	0
Unidentified Culicidae		33	17	50	76	84	160

^a^Only males of the *Ae. annulipes* group were identified to species level

### Effects of the resting site height

**Fig. 2:**
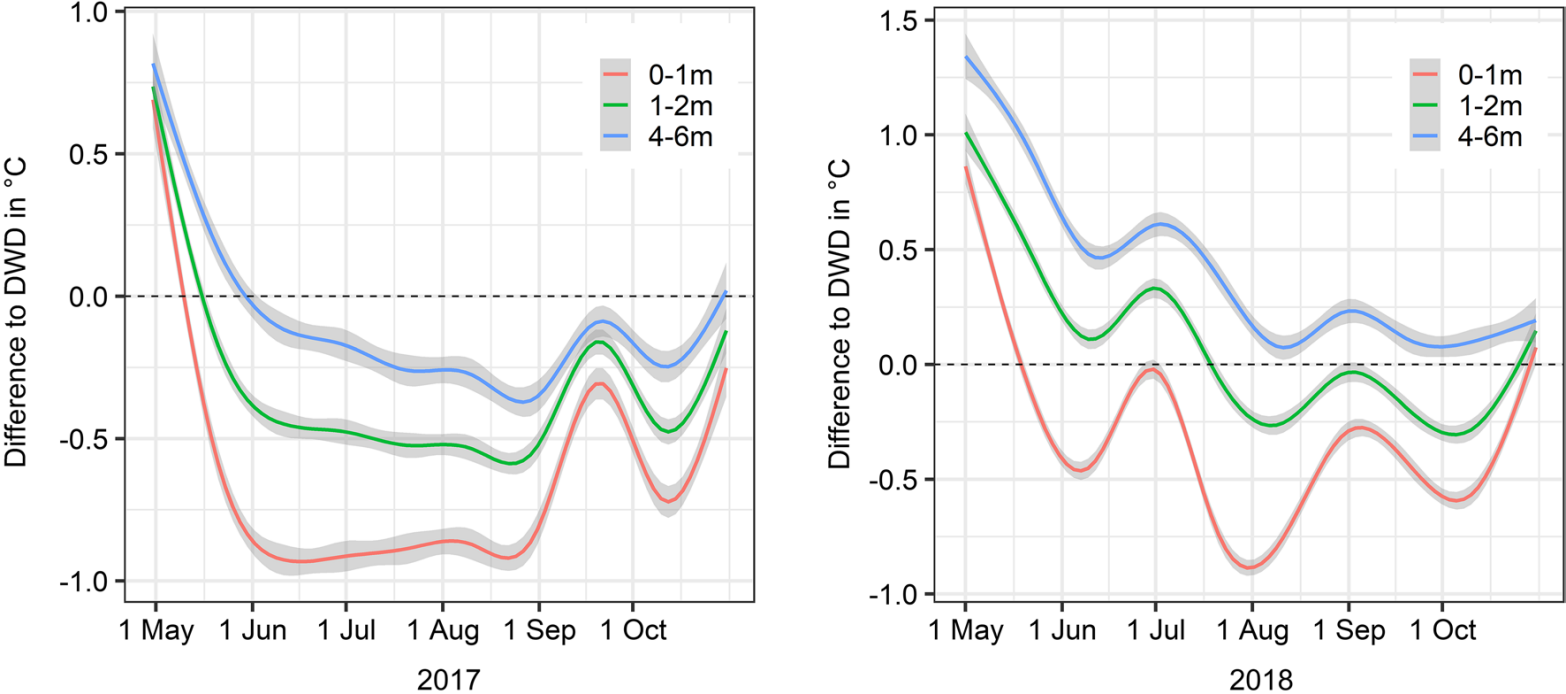
Temperature differences relative to the DWD data given for three analysed height levels. The temperature data is smoothed by general additive models, grey indicates the 99% confidence interval

**Fig. 3:**
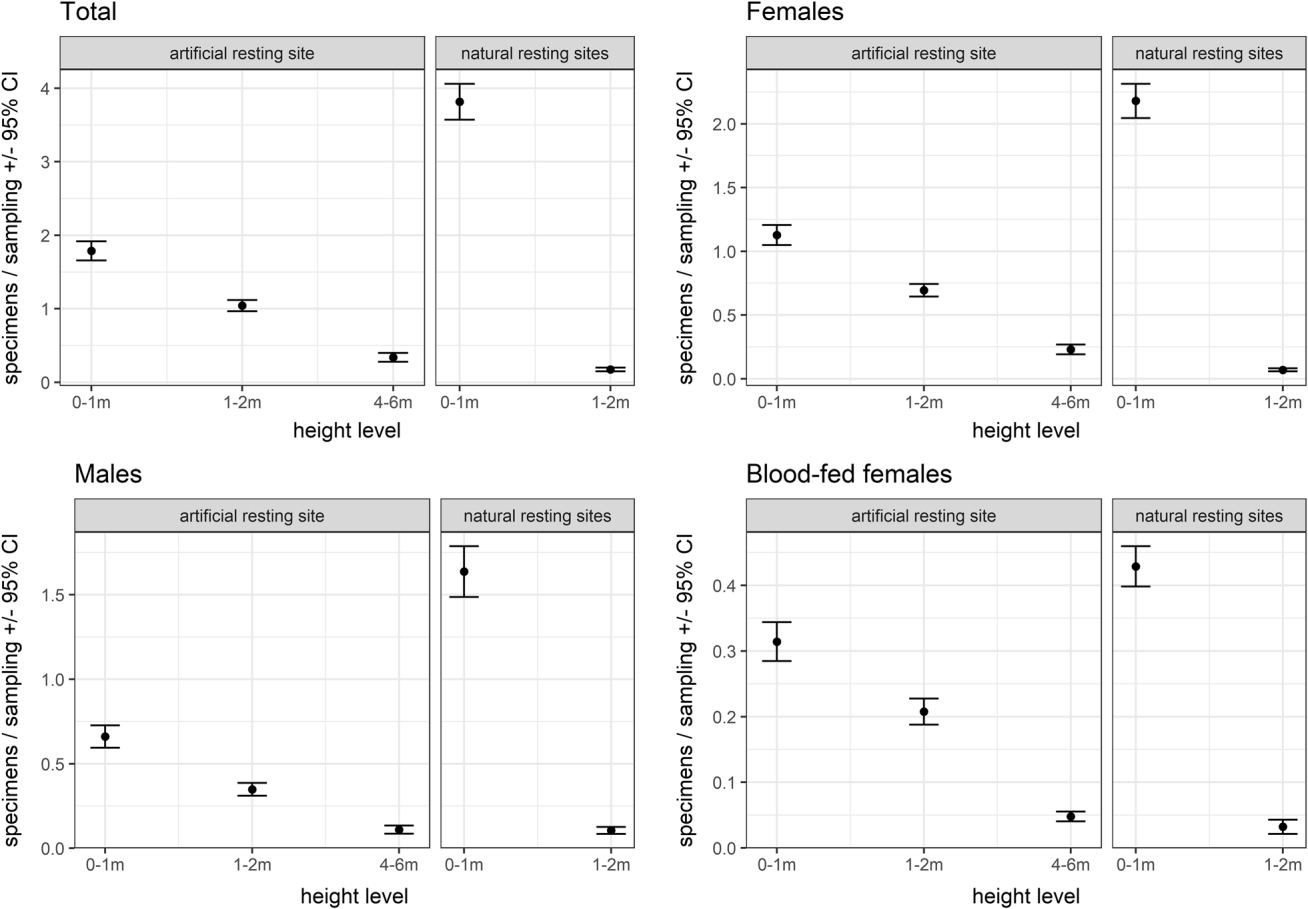
Mean number of mosquitoes with 95% confidence interval given for the resting site height and type of the total number of mosquitoes (7363 specimens), females (4440 specimens), males (2923 specimens) and blood-fed females (1070 specimens). Resting site collection data are summarized across both study years: 2017 and 2018

**Fig. 4:**
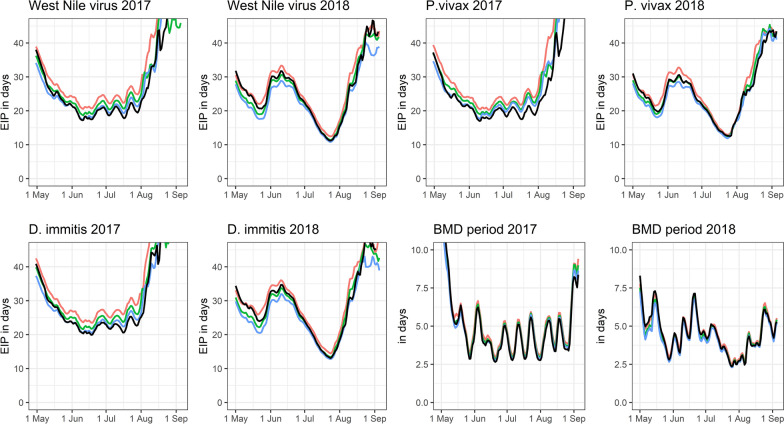
Mean EIP for different mosquito-borne pathogens and for the duration of the blood meal digestion (BMD) period calculated from the hourly microclimatic temperatures at different height levels (red: 0–1 m, green: 1–2 m, blue: 4–6 m) and the DWD temperatures in black

Overall, the mean temperatures were lower near the ground (0–1 m: mean = 16.23 °C ± 0.02 °C, 1–2 m: mean = 16.42 °C ± 0.02 °C, 4–6 m: mean = 16.70 °C ± 0.03 °C). In comparison with the DWD data, the temperatures were lower at heights between 0 and 2 m, but similar (in 2017) or even higher (in 2018) at elevated sites (4–6 m) (Fig. 2). At the beginning and at the end of both study periods, the microclimatic temperature differences between the three height levels converged and tended to be higher relative to the DWD data (Fig. 2). Irrespective of the resting site type (natural or artificial), higher numbers of mosquito specimens were collected at lower heights (0–2 m) (Fig. 3). There was no indication that females, males or blood-fed females have different resting height preferences (Fig. 3). Referring to species-specific height preferences, *Coquillettidia richiardii* was the only species without clear resting height preferences and was collected in similar numbers at all heights (Additional file 1: Table S2). All other taxa that were collected in sufficient numbers, were collected in higher numbers at resting sites between 0 and 2 m (Additional file 1: Table S2). The generally lower temperatures near the ground (Fig. 2) resulted in a prolonged EIP and blood meal digestion period compared with the calculation based on DWD temperatures (Figs. 4, 7).

### Effects of the land use

**Fig. 5:**
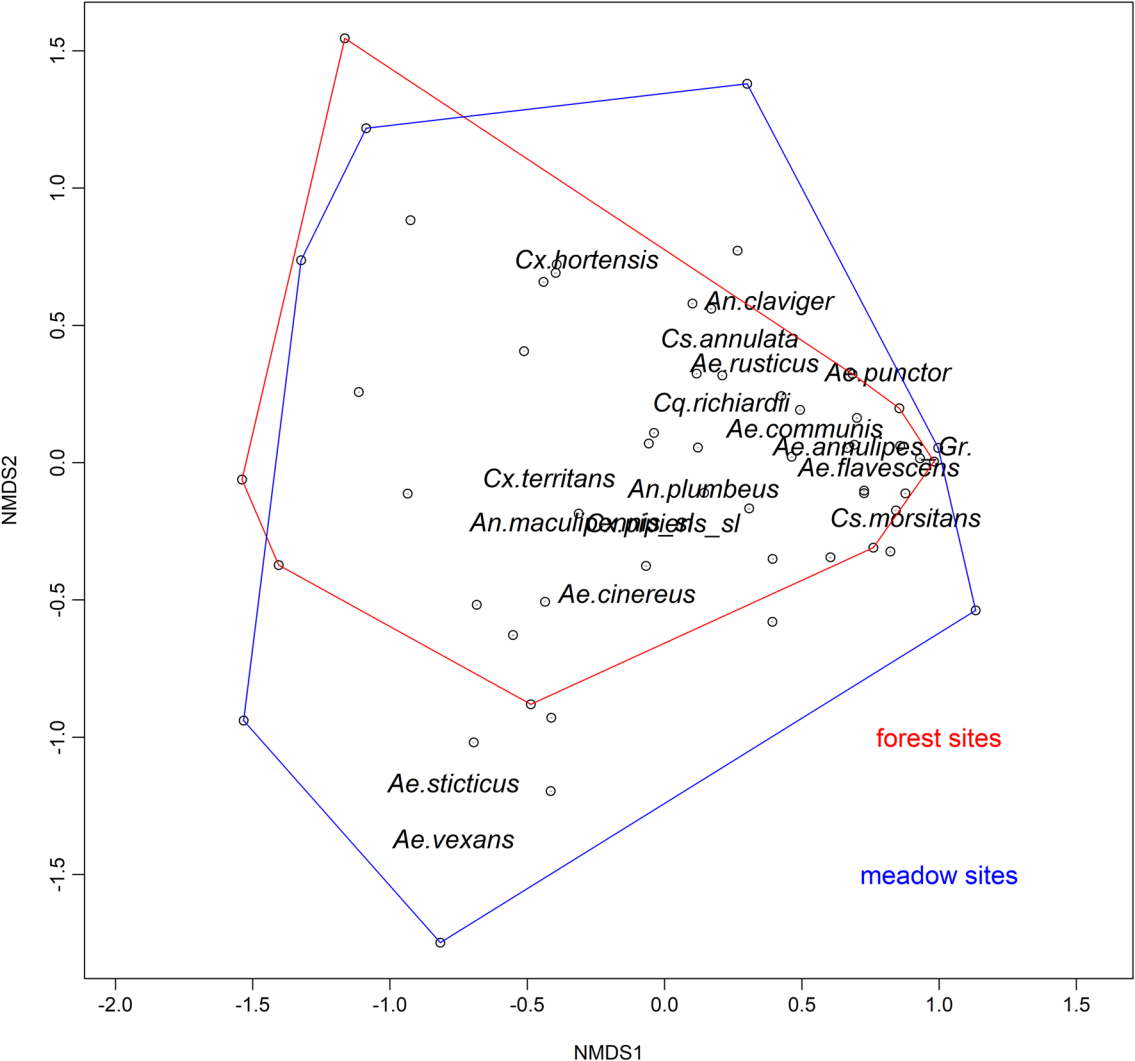
NMDS ordination of the mosquito species collected at meadow sites (framed in blue) and forest sites (framed in red) (Distance: Bray–Curtis, Dimension: 3, Stress 0.14). The labels of *An. maculipennis* s.l. and *Cx. pipiens* s.l. strongly overlap

**Fig. 6:**
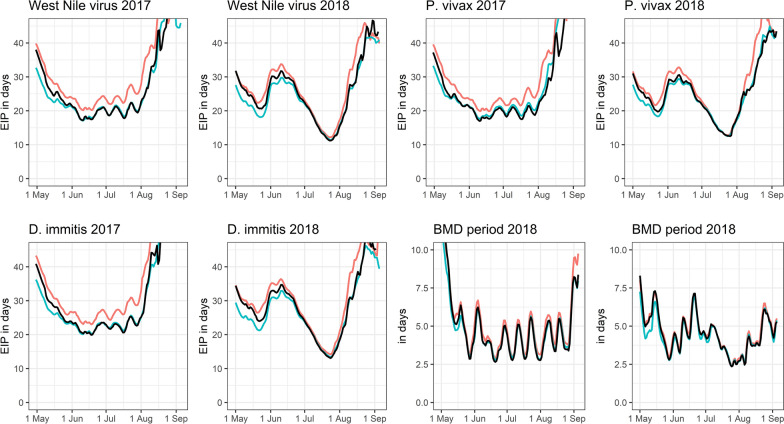
Mean EIP for 3 different mosquito-borne pathogens and for the duration of the blood meal digestion (BMD) period calculated from the microclimatic temperatures at different reclassified land use categories (red: forest, blue: meadows) and the DWD temperatures in black

**Fig. 7:**
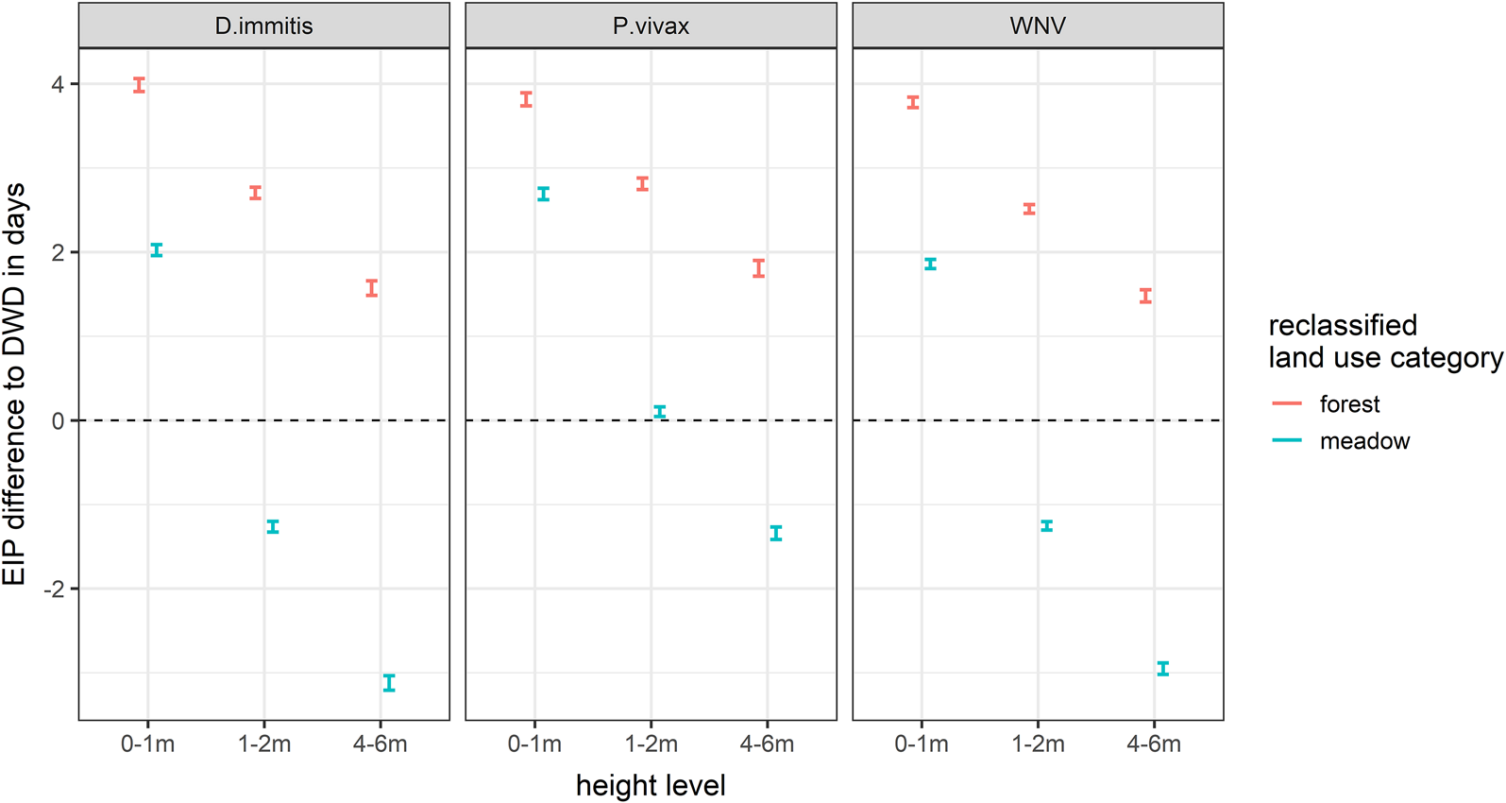
The 99% confidence intervals show the theoretic EIP of mosquito-borne pathogens based on the microclimatic temperatures at different heights and land use categories (red: forest, blue: meadows) relative to the EIP estimations based on the temperature data obtained from the DWD. The data include both study years: 2017 and 2018

Microclimatic temperatures were colder for the forest than for meadow sites: 15.83 ± 0.03 °C vs. 16.28 ± 0.03 °C in the study period 2017 and 16.56 ± 0.03 °C vs. 16.95 ± 0.03 °C in the study period 2018. The mosquito species composition did not differ significantly between the reclassified land use categories forest and meadows (Adonis, R^2^ = 0.03, p = 0.11), i.e. most species were found in both land use categories (Fig. 5). An exception was *Ae. vexans* and *Aedes sticticus*, which were rather associated with meadow sites (Fig. 5). The microclimate at meadow sites would have led to a similar duration of the EIP compared with the calculations based on the DWD temperatures (Fig. 6). Considering the height-specific temperature differences, the estimated mean EIPs at the forest sites were around 4 days longer based on the temperatures near the ground and 2 days longer based on temperatures at a height of 4–6 m. At meadow sites, the temperatures near the ground led to a prolonged EIP of 2 days, while temperatures at a height of 4–6 m would shorten the mean EIP up to 3 days relative to the calculations based on the DWD temperatures (Fig. 7).

## Discussion

Through the combination of active collection of resting mosquitoes and the associated temperatures, we demonstrate that the difference between standard meteorological data and resting site temperatures can affect the estimation of EIPs. In addition, we could confirm the high accuracy of the multiple linear regression approach by Haider et al. [30], which shows that it is possible to predict microclimatic temperatures based on standard meteorological data. The slightly higher accuracy for the microclimatic temperatures in the reclassified forest category in our study likely indicates that this category describes a more homogenous microclimatic landscape than the meadow category.

Considering mosquito preferences for resting sites near the ground in a shaded environment, mosquitoes experience microclimatic temperatures, which would prolong the EIP in near-natural landscapes beyond what is predicted by data from weather stations. Thus, resting site selection and resting behaviour can change the estimated duration of the EIP of mosquito-borne pathogens. In line with studies from North America e.g. [47], mosquito taxa prefer to rest either in cavities (e.g. pop-up bags or deadwood habitats) or in the vegetation. As other artificial resting sites [48, 49], garden pop-up bags are particularly useful for collecting cavity-resting genera *Anopheles*, *Culiseta* and *Culex*. *Aedes* are underrepresented in garden pop-up bags [25, 33]. Instead, we frequently found *Aedes* in natural resting habitats, especially in the herb layer, which is in accord with previous studies [22, 23, 50]. Irrespective of the type of resting habitat, mosquitoes distinctly prefer to rest near the ground. At first glance, this seems to contradict the observations of studies collecting mosquitoes with baited traps, which documented high numbers of several mosquito species at heights of 5 m and higher; in particular, ornithophilic mosquitoes are considered to seek their avian hosts at a greater height [51–53]. However, resting site selection is probably triggered by other environmental cues, e.g. dark contrasts as visual cues or a certain microclimate [22, 54, 55]. Hence, conclusions about the height preferences based on baited trapping methods focussing on host-seeking females are probably not transferable to mosquito resting height patterns.

Apart from the resting height preferences, the resting behaviour needs further investigation, in particular the mean resting duration and movement between resting sites are not well understood. Artificial resting site studies from North America suggest that mosquitoes enter and leave resting sites within a couple of hours before and after dawn, respectively. However, their conclusion relies on a few species and a few sampling days [56, 57]. In a previous resting site study using garden pop-up bags, mosquitoes were collected twice a day, whereby a higher number of mosquitoes were collected in the evening than in the morning, indicating that mosquitoes must have entered the resting sites during the day [33]. More detailed knowledge on resting duration, movement between resting sites and circadian rhythm of resting and activity periods would provide information on when and how much of mosquitoes’ lifetime is spent in their resting habitats. This, in turn, would help to adequately assess the relevance of the resting site microclimate for mosquito life history traits (e.g. adult survival) and the pathogen development rate in the mosquito vectors.

The observed effect of land use on the mosquito species composition was low. Most mosquito taxa were found in similar numbers in forests and meadows. The exceptions were *Ae. vexans* and *Ae. sticticus*, being associated with ecological parameters represented by the reclassified category meadow. These two species are particularly adapted to breeding sites in floodplain areas [58]. However, a mosaic of arable land, natural grassland, and softwood and hardwood floodplain forest in the different study areas in combination with the size-dependent thresholds for delineated land-use classification in CLC (e.g. CLC 231, pastures), floodplains are commonly grouped in the meadow category. Other mosquito taxa did not show clear patterns related to land use categories. This might be surprising at first sight, as mosquitoes are assumed to have a species-specific egg-laying behaviour in either forests or open areas [59]. However, the adult resting site collections could only partially confirm these patterns. As demonstrated by Bidlingmayer and Hem [60], some species seek breeding habitats in open areas but rest in a shaded environment in the forest, i.e. mosquitoes do not necessarily rest where they lay their eggs. Resting site collections reflect the adult mosquitoes, which emerge from varying types of breeding habitats in the larger radius of the sampling sites. These aspects probably complicate the establishment of a clear relationship between land use factors and their associated microclimate and the mosquito species composition when focussing on near-natural landscapes with a presumably high diversity of different breeding habitats.

## Conclusions

Understanding the microclimatic conditions of mosquito resting sites can aid in refining epidemiological models. This study showed that the colder temperatures at mosquito preferred resting sites near the ground would prolong the EIP of mosquito-borne pathogens relative to standardized temperature data from weather stations. However, our results are restricted to a near-natural environment. Future studies should investigate mosquito resting site patterns and associated microclimates in more habitat types and environments to analyse their relevance to the spatial risk of mosquito-borne pathogen transmission.

## Supplementary Information

**Additional file 1: Table S1.** Results of the multiple linear regressions to predict the resting site temperatures in the two habitats meadows and forest for both study periods. **Table S2.** Mean number (with 95% confidence interval) of specimens per species collected per resting site type and height level. Total number resting site sampling events: 5043 (3213 in artificial resting sites and 1830 in natural resting sites).

## Data Availability

The datasets and statistical analysis for the current study are available from the corresponding authors on reasonable request.
