# Supplementary material for: Effects of mosquito resting site temperatures on the estimation of pathogen development rates in near-natural habitats in Germany
Source: Parasit Vectors. 2022 Oct 25;15:390. doi: 10.1186/s13071-022-05505-2 (PMC9594938; doi:10.1186/s13071-022-05505-2)
Supplement: Supplementary file 1 — Additional file 1: Table S1. Results of the multiple linear regressions to predict the resting site temperatures in the two habitats meadows and forest for both study periods. Table S2. Mean number (with 95% confidence interval) of specimens per species collected per resting site type and height level. Total number resting site sampling events: 5043 (3213 in artificial resting sites and 1830 in natural resting sites). [file 13071_2022_5505_MOESM1_ESM.docx]

**Supplementary data**

**Effects of mosquito resting site temperatures on the estimation of pathogen development rates in near-natural habitats in Germany**

Sauer, Felix Gregor^1^, Kiel, Ellen^2^, Lühken, Renke^1^

^1^Arbovirus Ecology, Department of Arbovirology, Bernhard Nocht Institute for Tropical Medicine, Hamburg, Germany

^2^Aquatic Ecology and Nature Conservation, Carl von Ossietzky University, Oldenburg, Germany

Correspondence: felix.sauer@bnitm.de

Keywords: Culicidae, microclimate, resting sites, Extrinsic Incubation Period

Table S1: Results of the multiple linear regressions to predict the resting site temperatures in the two habitats meadows and forest for both study periods.

|  | **Meadow sites 2017** | | | | **Forest sites 2017** | | | | **Meadow sites 2018** | | | | **Forest sites 2018** | | | |
| --- | --- | --- | --- | --- | --- | --- | --- | --- | --- | --- | --- | --- | --- | --- | --- | --- |
| **factors** | **coefficient** | **Std. Error** | **t-value** | **p value** | **coefficient** | **Std. Error** | **t-value** | **p value** | **coefficient** | **Std. Error** | **t-value** | **p value** | **coefficient** | **Std. Error** | **t-value** | **p value** |
| (Intercept) | -6,44E+03 | 1,04E+03 | -6.210 | 5.31e-10 | -3,85E+03 | 6,02E+02 | -6.400 | 1.55e-10 | 1,18E+04 | 9,29E+02 | 12.677 | < 2e-16 | 2,99E+04 | 9,20E+02 | 32.548 | < 2e-16 |
| Temperature DWD | -1,38E+02 | 5,09E+00 | -27.080 | < 2e-16 | -1,89E+01 | 3,01E+00 | -6.288 | 3.21e-10 | 8,88E+02 | 1,35E+00 | 655.415 | < 2e-16 | 1,05E+02 | 3,88E+00 | 27.005 | < 2e-16 |
| Temperature DWD (t-1) | 1,05E+03 | 4,87E+00 | 214.646 | < 2e-16 | 9,04E+02 | 2,88E+00 | 314.067 | < 2e-16 | 1,83E+01 | 5,43E-01 | 33.683 | < 2e-16 | 7,48E+02 | 3,72E+00 | 200.864 | < 2e-16 |
| Radiation | 3,98E+00 | 4,24E-01 | 9.401 | < 2e-16 | 2,91E+00 | 2,75E-01 | 10.583 | < 2e-16 | -4,40E+00 | 1,62E-01 | -27.213 | < 2e-16 | 3,79E+00 | 1,48E-01 | 25.653 | < 2e-16 |
| Wind | -8,33E+01 | 3,88E+00 | -21.456 | < 2e-16 | -6,96E+00 | 2,78E+00 | -2.500 | 0.0124 | 1,93E+01 | 3,11E+00 | 6.207 | 5.40e-10 | 4,58E+01 | 2,07E+00 | 22.137 | < 2e-16 |
| Humidity DWD | 8,84E+00 | 4,93E-01 | 17.925 | < 2e-16 | 1,81E+01 | 2,81E-01 | 64.391 | < 2e-16 | 2,50E+01 | 3,52E-01 | 71.024 | < 2e-16 | 1,79E+01 | 3,62E-01 | 49.406 | < 2e-16 |
| Month4 | 5,69E+03 | 1,01E+03 | 5.664 | 1.48e-08 | 3,12E+03 | 5,84E+02 | 5.345 | 9.03e-08 | -1,14E+04 | 8,92E+02 | -12.769 | < 2e-16 | -2,88E+04 | 8,83E+02 | -32.619 | < 2e-16 |
| Month5 | 6,78E+03 | 1,04E+03 | 6.529 | 6.64e-11 | 3,48E+03 | 6,04E+02 | 5.761 | 8.36e-09 | -1,21E+04 | 9,20E+02 | -13.170 | < 2e-16 | -2,90E+04 | 9,10E+02 | -31.898 | < 2e-16 |
| Month6 | 7,00E+03 | 1,04E+03 | 6.748 | 1.50e-11 | 3,94E+03 | 6,03E+02 | 6.533 | 6.47e-11 | -1,24E+04 | 9,18E+02 | -13.506 | < 2e-16 | -2,91E+04 | 9,09E+02 | -32.016 | < 2e-16 |
| Month7 | 6,96E+03 | 1,03E+03 | 6.748 | 1.51e-11 | 3,88E+03 | 5,99E+02 | 6.465 | 1.01e-10 | -1,21E+04 | 9,12E+02 | -13.295 | < 2e-16 | -2,82E+04 | 9,03E+02 | -31.275 | < 2e-16 |
| Month8 | 6,68E+03 | 1,04E+03 | 6.424 | 1.33e-10 | 3,73E+03 | 6,04E+02 | 6.169 | 6.86e-10 | -1,24E+04 | 9,19E+02 | -13.499 | < 2e-16 | -2,85E+04 | 9,10E+02 | -31.297 | < 2e-16 |
| Month9 | 6,35E+03 | 1,04E+03 | 6.114 | 9.74e-10 | 3,28E+03 | 6,03E+02 | 5.428 | 5.70e-08 | -1,31E+04 | 9,19E+02 | -14.237 | < 2e-16 | -2,95E+04 | 9,10E+02 | -32.438 | < 2e-16 |
| Month10 | 5,83E+03 | 1,03E+03 | 5.658 | 1.53e-08 | 3,04E+03 | 5,98E+02 | 5.083 | 3.71e-07 | -1,34E+04 | 9,13E+02 | -14.691 | < 2e-16 | -2,97E+04 | 9,04E+02 | -32.891 | < 2e-16 |
| Month11 | 5,77E+03 | 1,05E+03 | 5.493 | 3.97e-08 | 2,58E+03 | 6,11E+02 | 4.220 | 2.44e-05 | -1,40E+04 | 9,35E+02 | -15.000 | < 2e-16 | -3,20E+04 | 9,25E+02 | -34.542 | < 2e-16 |
| Hour1 | -8,76E+01 | 3,04E+01 | -2.878 | 0.0040 | -1,32E+02 | 1,77E+01 | -7.469 | 8.13e-14 | -9,63E+01 | 2,47E+01 | -3.893 | 9.90e-05 | 6,17E+00 | 2,46E+01 | 0.250 | 0.8023 |
| Hour2 | -1,74E+02 | 3,04E+01 | -5.715 | 1.10e-08 | -2,51E+02 | 1,77E+01 | -14.183 | < 2e-16 | -2,17E+02 | 2,47E+01 | -8.776 | < 2e-16 | -4,51E+01 | 2,47E+01 | -1.827 | 0.0677 |
| Hour3 | -2,22E+02 | 3,05E+01 | -7.288 | 3.15e-13 | -3,07E+02 | 1,77E+01 | -17.305 | < 2e-16 | -2,85E+02 | 2,48E+01 | -11.482 | < 2e-16 | -1,03E+02 | 2,47E+01 | -4.185 | 2.85e-05 |
| Hour4 | -2,61E+02 | 3,05E+01 | -8.558 | < 2e-16 | -3,44E+02 | 1,78E+01 | -19.375 | < 2e-16 | -5,16E+02 | 2,48E+01 | -20.784 | < 2e-16 | -1,65E+02 | 2,47E+01 | -6.680 | 2.40e-11 |
| Hour5 | -2,57E+02 | 3,08E+01 | -8.356 | < 2e-16 | -3,52E+02 | 1,79E+01 | -19.625 | < 2e-16 | -1,09E+03 | 2,49E+01 | -43.877 | < 2e-16 | -4,42E+02 | 2,51E+01 | -17.594 | < 2e-16 |
| Hour6 | -6,35E+02 | 3,15E+01 | -20.201 | < 2e-16 | -7,64E+02 | 1,83E+01 | -41.797 | < 2e-16 | -1,48E+03 | 2,55E+01 | -58.277 | < 2e-16 | -9,95E+02 | 2,57E+01 | -38.646 | < 2e-16 |
| Hour7 | -6,99E+02 | 3,24E+01 | -21.568 | < 2e-16 | -1,17E+03 | 1,89E+01 | -62.295 | < 2e-16 | -1,49E+03 | 2,65E+01 | -56.068 | < 2e-16 | -1,42E+03 | 2,66E+01 | -53.258 | < 2e-16 |
| Hour8 | -2,77E+02 | 3,38E+01 | -8.203 | 2.36e-16 | -1,17E+03 | 1,96E+01 | -59.306 | < 2e-16 | -1,10E+03 | 2,80E+01 | -39.146 | < 2e-16 | -1,45E+03 | 2,79E+01 | -51.797 | < 2e-16 |
| Hour9 | 4,16E+02 | 3,52E+01 | 11.823 | < 2e-16 | -7,48E+02 | 2,05E+01 | -36.503 | < 2e-16 | -4,97E+02 | 2,96E+01 | -16.806 | < 2e-16 | -1,35E+03 | 2,92E+01 | -46.318 | < 2e-16 |
| Hour10 | 1,15E+03 | 3,63E+01 | 31.598 | < 2e-16 | -1,13E+02 | 2,12E+01 | -5.343 | 9.17e-08 | 1,62E+02 | 3,08E+01 | 5.277 | 1.31e-07 | -9,46E+02 | 3,03E+01 | -31.167 | < 2e-16 |
| Hour11 | 1,53E+03 | 3,71E+01 | 41.313 | < 2e-16 | 4,76E+02 | 2,16E+01 | 21.980 | < 2e-16 | 7,35E+02 | 3,13E+01 | 23.475 | < 2e-16 | -5,06E+02 | 3,10E+01 | -16.328 | < 2e-16 |
| Hour12 | 1,89E+03 | 3,71E+01 | 50.898 | < 2e-16 | 9,36E+02 | 2,16E+01 | 43.288 | < 2e-16 | 1,27E+03 | 3,11E+01 | 40.745 | < 2e-16 | -2,23E+02 | 3,09E+01 | -7.204 | 5.86e-13 |
| Hour13 | 2,10E+03 | 3,61E+01 | 58.068 | < 2e-16 | 1,34E+03 | 2,10E+01 | 63.518 | < 2e-16 | 1,64E+03 | 3,04E+01 | 53.881 | < 2e-16 | 1,34E+02 | 3,01E+01 | 4.464 | 8.03e-06 |
| Hour14 | 2,22E+03 | 3,50E+01 | 63.510 | < 2e-16 | 1,42E+03 | 2,04E+01 | 69.582 | < 2e-16 | 1,84E+03 | 2,92E+01 | 63.104 | < 2e-16 | 4,09E+02 | 2,89E+01 | 14.147 | < 2e-16 |
| Hour15 | 2,37E+03 | 3,37E+01 | 70.397 | < 2e-16 | 1,38E+03 | 1,97E+01 | 69.804 | < 2e-16 | 1,91E+03 | 2,79E+01 | 68.534 | < 2e-16 | 5,26E+02 | 2,78E+01 | 18.954 | < 2e-16 |
| Hour16 | 2,56E+03 | 3,27E+01 | 78.243 | < 2e-16 | 1,21E+03 | 1,91E+01 | 63.296 | < 2e-16 | 2,07E+03 | 2,68E+01 | 77.109 | < 2e-16 | 5,24E+02 | 2,67E+01 | 19.623 | < 2e-16 |
| Hour17 | 2,54E+03 | 3,21E+01 | 79.214 | < 2e-16 | 1,03E+03 | 1,87E+01 | 54.997 | < 2e-16 | 2,11E+03 | 2,61E+01 | 80.633 | < 2e-16 | 5,72E+02 | 2,60E+01 | 22.005 | < 2e-16 |
| Hour18 | 2,30E+03 | 3,17E+01 | 72.464 | < 2e-16 | 8,96E+02 | 1,85E+01 | 48.446 | < 2e-16 | 2,07E+03 | 2,56E+01 | 80.911 | < 2e-16 | 4,91E+02 | 2,56E+01 | 19.135 | < 2e-16 |
| Hour19 | 1,80E+03 | 3,13E+01 | 57.525 | < 2e-16 | 8,23E+02 | 1,83E+01 | 44.964 | < 2e-16 | 1,93E+03 | 2,52E+01 | 76.504 | < 2e-16 | 2,63E+02 | 2,54E+01 | 10.350 | < 2e-16 |
| Hour20 | 1,34E+03 | 3,09E+01 | 43.330 | < 2e-16 | 7,30E+02 | 1,80E+01 | 40.463 | < 2e-16 | 1,44E+03 | 2,49E+01 | 57.695 | < 2e-16 | 1,92E+02 | 2,50E+01 | 7.690 | 1.47e-14 |
| Hour21 | 7,56E+02 | 3,06E+01 | 24.719 | < 2e-16 | 6,08E+02 | 1,78E+01 | 34.122 | < 2e-16 | 8,12E+02 | 2,48E+01 | 32.708 | < 2e-16 | 1,38E+02 | 2,48E+01 | 5.572 | 2.52e-08 |
| Hour22 | 2,70E+02 | 3,05E+01 | 8.845 | < 2e-16 | 2,94E+02 | 1,77E+01 | 16.551 | < 2e-16 | 3,66E+02 | 2,48E+01 | 14.789 | < 2e-16 | 5,17E+01 | 2,47E+01 | 2.097 | 0.0360 |
| Hour23 | 6,10E+01 | 3,04E+01 | 2.004 | 0.0451 | 1,35E+02 | 1,77E+01 | 7.612 | 2.71e-14 | 1,44E+02 | 2,47E+01 | 5.828 | 5.60e-09 | -2,04E-01 | 2,47E+01 | -0.008 | 0.9934 |
| radiation:wind | 3,52E-01 | 2,75E-02 | 12.796 | < 2e-16 | 7,73E-01 | 1,86E-02 | 41.532 | < 2e-16 | 8,87E-01 | 2,42E-02 | 36.620 | < 2e-16 | 3,14E-01 | 1,83E-02 | 17.190 | < 2e-16 |
| humidity_DWD:precipitation | 8,30E-01 | 7,62E-02 | 10.898 | < 2e-16 | 8,95E-01 | 4,53E-02 | 19.776 | < 2e-16 | -9,34E-03 | 1,83E-03 | -5.118 | 3.09e-07 | -4,47E-03 | 1,10E-03 | -4.060 | 4.91e-05 |
| radiation:as.factor(month)5 | -3,04E+00 | 4,02E-01 | -7.572 | 3.69e-14 | -5,86E+00 | 2,63E-01 | -22.301 | < 2e-16 | NA | NA | NA | NA | NA | NA | NA | NA |
| radiation:as.factor(month)6 | -6,55E+00 | 3,97E-01 | -16.503 | < 2e-16 | -9,09E+00 | 2,60E-01 | -34.983 | < 2e-16 | -3,40E+00 | 1,16E-01 | -29.214 | < 2e-16 | -3,59E+00 | 1,15E-01 | -31.309 | < 2e-16 |
| radiation:as.factor(month)7 | -8,71E+00 | 4,04E-01 | -21.561 | < 2e-16 | -1,03E+01 | 2,64E-01 | -39.233 | < 2e-16 | -3,47E+00 | 1,11E-01 | -31.390 | < 2e-16 | -3,75E+00 | 1,10E-01 | -34.275 | < 2e-16 |
| radiation:as.factor(month)8 | -7,23E+00 | 4,09E-01 | -17.694 | < 2e-16 | -1,07E+01 | 2,66E-01 | -40.155 | < 2e-16 | -3,76E+00 | 1,23E-01 | -30.503 | < 2e-16 | -4,63E+00 | 1,23E-01 | -37.550 | < 2e-16 |
| radiation:as.factor(month)9 | -8,10E+00 | 4,24E-01 | -19.116 | < 2e-16 | -1,23E+01 | 2,74E-01 | -45.036 | < 2e-16 | -2,70E+00 | 1,49E-01 | -18.056 | < 2e-16 | -3,88E+00 | 1,50E-01 | -25.902 | < 2e-16 |
| radiation:as.factor(month)10 | -7,87E+00 | 4,47E-01 | -17.609 | < 2e-16 | -1,35E+01 | 2,87E-01 | -46.800 | < 2e-16 | -2,94E+00 | 2,09E-01 | -14.042 | < 2e-16 | -4,09E+00 | 2,09E-01 | -19.613 | < 2e-16 |
| wind:as.factor(height)2 | 8,58E+01 | 3,66E+00 | 23.429 | < 2e-16 | 4,12E+01 | 2,52E+00 | 16.346 | < 2e-16 | 6,47E+01 | 2,66E+00 | 24.283 | < 2e-16 | 2,92E+01 | 1,99E+00 | 14.708 | < 2e-16 |
| wind:as.factor(height)3 | 1,49E+02 | 3,99E+00 | 37.373 | < 2e-16 | 5,73E+01 | 2,87E+00 | 19.975 | < 2e-16 | 9,20E+00 | 3,17E+00 | 2.898 | 0.00376 | 1,38E+01 | 2,40E+00 | 5.724 | 1.04e-08 |
| radiation:as.factor(height)2 | 2,24E+00 | 1,12E-01 | 20.058 | < 2e-16 | 1,33E+00 | 6,76E-02 | 19.711 | < 2e-16 | 2,38E+00 | 8,97E-02 | 26.578 | < 2e-16 | 1,70E+00 | 8,28E-02 | 20.524 | < 2e-16 |
| radiation:as.factor(height)3 | 1,84E+00 | 1,23E-01 | 14.918 | < 2e-16 | 2,65E+00 | 7,61E-02 | 34.865 | < 2e-16 | 1,06E+01 | 1,07E-01 | 98.532 | < 2e-16 | 6,70E+00 | 1,00E-01 | 66.798 | < 2e-16 |
| Summary | Degrees of freedom: 225580 | | | | Degrees of freedom: 323988 | | | | Degrees of freedom: 355098 | | | | Degrees of freedom: 235230 | | | |
|  | R-squared: 0.893 | | | | R-squared: 0.932 | | | | R-squared: 0.8913 | | | | R-squared: 0.910 | | | |
|  | F-statistic: 3.913e+04 | | | | F-statistic: 9.244e+04 | | | | F-statistic: 6.194e+04 | | | | F-statistic: 5.069e+04 | | | |
|  | p-value: < 2.2e-16 | | | | p-value: < 2.2e-16 | | | | p-value: < 2.2e-16 | | | | p-value: < 2.2e-16 | | | |

Table S2: Mean number (with 95% confidence interval) of specimens per species collected per resting site type and height level. Total number resting site sampling events: 5043 (3213 in artificial resting sites and 1830 in natural resting sites).

| **Mosquito taxon** | **Artificial resting sites** | | | **Natural resting sites** | |
| --- | --- | --- | --- | --- | --- |
|  | 0-1 m | 1-2 m | 4-6 m | 0-2 m | 2-6 m |
| *Ae. annulipes* group | 0.1208 (0.0321) | 0.0365 (0.0139) | 0.0134 (0.007) | 2.0464 (0.3893) | 0.0536 (0.0239) |
| *Ae. vexans* | 0.0209 (0.0166) | 0.0019 (0.0026) | 0.001 (0.0019) | 0.5459 (0.1833) | 0.031 (0.0171) |
| *Ae. cinereus* | 0.0073 (0.0067) | 0.0037 (0.0037) | 0.001 (0.0019) | 0.2069 (0.0522) | 0.0048 (0.0047) |
| *Ae. rusticus* | 0.0045 (0.0047) | 0.0084 (0.0076) | 0 | 0.0989 (0.0442) | 0 |
| *Ae. sticticus* | 0.010 (0.0069) | 0.0075 (0.0058) | 0.0029 (0.0033) | 0.1171 (0.0558) | 0.0238 (0.0206) |
| *Ae. punctor* | 0.0064 (0.0047) | 0.0019 (0.0026) | 0.0019 (0.0027) | 0.0827 (0.0502) | 0.006 (0.0096) |
| *An. maculipennis* s.l. | 0.1199 (0.0269) | 0.0870 (0.0273) | 0.0125 (0.0073) | 0.005 (0.0044) | 0 |
| *Cq. richiardii* | 0.0036 (0.0036) | 0.0094 (0.0100) | 0.0077 (0.0059) | 0.0131 (0.0071) | 0.0167 (0.0227) |
| *Cs. morsitans*/*fumipennis* | 1.0745 (0.2473) | 0.5996 (0.1282) | 0.2263 (0.1175) | 0.1514 (0.0687) | 0.0036 (0.004) |
| *Cs. annulata*/*subochrea* | 0.1126 (0.0291) | 0.1188 (0.0301) | 0.0153 (0.0084) | 0.0383 (0.0152) | 0.0036 (0.004) |
| *Cx. pipiens* s.l./*Cx. torrentium* | 0.1962 (0.0424) | 0.0645 (0.0216) | 0.0249 (0.0118) | 0.2099 (0.0592) | 0.0024 (0.0033) |
| *Cx. territans* | 0.0581 (0.0175) | 0.0571 (0.0178) | 0.0163 (0.0077) | 0.0313 (0.0131) | 0.006 (0.0062) |
